# Surface Sampling Methods for *Bacillus anthracis* Spore Contamination

**DOI:** 10.3201/eid0810.020382

**Published:** 2002-10

**Authors:** Wayne T. Sanderson, Misty J. Hein, Lauralynn Taylor, Brian D. Curwin, Gregory M. Kinnes, Teresa A. Seitz, Tanja Popovic, Harvey T. Holmes, Molly E. Kellum, Sigrid K. McAllister, David N. Whaley, Edward A. Tupin, Timothy Walker, Jennifer A. Freed, Dorothy S. Small, Brian Klusaritz, John H. Bridges

**Affiliations:** * Centers for Disease Control and Prevention, Atlanta, Georgia, USA; † Agency for Toxic Substances and Disease Registry, Atlanta, Georgia, USA; ‡ The IT Corporation, Washington, D.C., USA; § United States Postal Service, Washington, D.C., USA

**Keywords:** Bacillus anthracis, anthrax, bacterial spores, surface sampling, HEPA vacuum sock, swabs, wipes, postal facility, bioterrorism

## Abstract

During an investigation conducted December 17–20, 2001, we collected environmental samples from a U.S. postal facility in Washington, D.C., known to be extensively contaminated with *Bacillus anthracis* spores. Because methods for collecting and analyzing *B. anthracis* spores have not yet been validated, our objective was to compare the relative effectiveness of sampling methods used for collecting spores from contaminated surfaces. Comparison of wipe, wet and dry swab, and HEPA vacuum sock samples on nonporous surfaces indicated good agreement between results with HEPA vacuum and wipe samples. However, results from HEPA vacuum sock and wipe samples agreed poorly with the swab samples. Dry swabs failed to detect spores >75% of the time they were detected by wipe and HEPA vacuum samples. Wipe samples collected after HEPA vacuum samples and HEPA vacuum samples after wipe samples indicated that neither method completely removed spores from the sampled surfaces.

The Brentwood Mail Processing and Distribution Center in Washington, D.C., was extensively contaminated with *Bacillus anthracis* spores after two letters containing spores were processed at this facility on October 12, 2001 (1). Subsequently, inhalational anthrax developed in four postal workers. An investigation in late October 2001, using surface wipe and HEPA vacuum sock sampling techniques, showed widespread *B. anthracis* spore contamination inside the building. Spore concentrations were particularly high around Delivery Bar Code Sorter (DBCS) machine no. 17, which had processed the letters, and in the government mail area, where the letters had been processed before being distributed.

This report describes the results of sampling for *B. anthracis* spores in an investigation conducted December 17–20, 2001, by the Centers for Disease Control and Prevention (CDC), the Agency for Toxic Substances and Disease Registry (ATSDR), the U.S. Postal Service (USPS), and a USPS contractor. At the time of this investigation, technical issues regarding sampling and analyses for *B. anthracis* spores remained unresolved, such as which technique for surface sampling (swabs, wipes, or HEPA vacuum socks) is most appropriate for collecting spores in specific situations, how the different types of surface sampling methods compare, and how effectively the sampling methods collect spores from contaminated surfaces. Surface sampling to determine the presence of *B. anthracis* spores in an environment is essential for determining extent of contamination, assessing potential for exposure and need for medical treatment, and guiding clean-up and reentry efforts.

Sampling methods (swabs, wipes, rinses, direct agar contact, and vacuuming) have been evaluated for collecting microorganisms from surfaces (2–7), primarily in laboratory settings. *B. subtilis* spores, which may behave much like *B. anthracis* spores, have been frequently used as the microbiologic agent sampled. Substantial variation in sample recoveries was observed for the various methods. In addition, the methods have not been validated specifically for collecting and analyzing *B. anthracis* spores in environmental samples. The primary objective of our survey was to compare the levels of *B. anthracis* spores in side-by-side samples obtained by the surface swab, wipe, and HEPA vacuum sock methods to evaluate their relative effectiveness.

USPS representatives and a USPS contractor had conducted clean-up operations at the Brentwood facility since late October. However, much of the facility had not been cleaned and was believed still contaminated with *B. anthracis* spores. Even though the DBCS machine (no. 17) that processed the contaminated letters had been cleaned by HEPA vacuum, washed with a 10% sodium hypochlorite solution followed by neutralization with a sodium thiosulfate solution, and rinsed with water, this machine was reportedly still contaminated with *B. anthracis* spores (8). For these reasons, the Brentwood facility was thought to be a good location to compare surface sampling and analytical methods.

## Methods

Surface sampling was conducted by using swabs, wipes, and HEPA vacuum socks. To compare the sampling techniques, we selected locations where the three types of samples could be collected adjacent to each other on nonporous surfaces, with an emphasis on locations believed to be still contaminated with *B. anthracis* spores. The locations sampled included the surfaces of selected DBCS machines (particularly machine no. 17), return air ducts, tops of the window boxes along the postal inspector walkways, and the tops of mail sorting bins in a secure area approximately 23 m from DBCS machine no. 17. The order in which the samples were collected varied in a randomized fashion from location to location; each site was assigned a location number and sampled according to a predetermined, randomized plan. We used the randomized sampling plan to reduce sampling biases that might be caused by nonuniform distribution of spores across surfaces and affected by the order in which samples were collected.

Seven swab, six wipe, and five HEPA vacuum sock samples were collected as control samples; that is, these samples were handled in the same way as others but not used to sample any surfaces. The purpose of these control samples was to evaluate the potential contamination of sample media, unrelated to actual sample collection.

Nine additional blank HEPA vacuum sock samples were collected to estimate cross-contamination by inserting them into the vacuum nozzle after a HEPA vacuum sock sample had been collected and the nozzle cleaned; these socks were then withdrawn and placed in a sterile conical tube for laboratory analysis.

**Figure 1:**
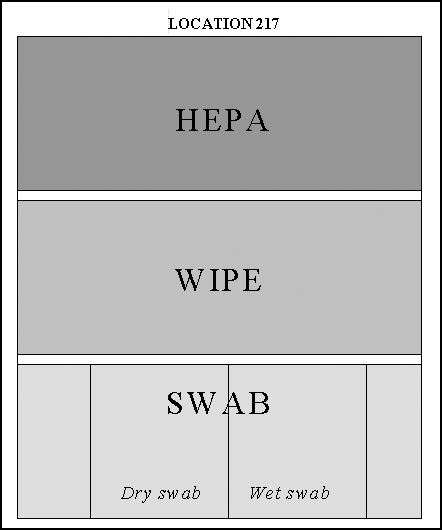
Sample instructions for collection of swab, wipe, and HEPA vacuum sock samples, Brentwood Mail Processing and Distribution Center, 2001. For specific location, investigator was given these instructions (exact text follows). Divide the selected space into three sections where each of the three types of surface samples (swab, wipe, HEPA vacuum sock) may be collected. Follow the random key above to designate which section will be sampled by each method and in which order the samples will be collected (follow top to bottom). Record the area of surface sampled by each method. The surface areas need not be equal, but should be sufficient to provide adequate sample collection for each method. Sample order for location was: 1) Collect the HEPA vacuum sock sample first and record surface area. After sampling, clean vacuum nozzle with alcohol and insert clean vacuum sock; remove this sock without sampling to serve as “contamination blank.” 2) Collect the WIPE sample second and record surface area. 3) Collect the SWAB samples third and record surface area. The first swab sample should be collected without moistening it. The second swab sample should be sampled pre-moistened. Take care not to overload swabs. 4) Collect an additional WIPE sample across the entire area which had been sampled by HEPA vacuum sock. 5).Collect an additional HEPA vacuum sock sample across the entire which had been sampled by WIPE.

Investigators were given written instructions for collecting samples at each selected location (Figure 1). The surface areas sampled by each technique were intended to be comparable, but not necessarily equal. In particularly dirty areas, swabs and wipes could not cover as large a surface area as the HEPA vacuum sock samples without becoming overloaded; investigators were instructed to avoid overloading the samples by adjusting the size of the surface sampled.

The following procedures, used to collect the three types of surface samples, were recommended for collecting surface environmental samples for culturing *B. anthracis* (9). The surface samples were all collected after investigators had donned nonpowdered gloves over two pairs of nitrile protective gloves, as part of the personal protective gear. The area of the surface sampled was measured with a tape measure and recorded in square centimeters.

Swab samples were collected by removing a sterile, rayon (noncotton) swab (Environmental Swab Kit, CDC, Atlanta, GA) from a sterile tube, moistening it by inserting it into a second tube which contained a sponge soaked with sterile 1.5 mL of phosphate-buffered saline (PBS) at pH 7.2, and then swabbing the selected surface by moving the swab back and forth across the surface with several horizontal strokes, then several vertical strokes. The swab was rotated during sampling to ensure that the entire surface of the swab was used. After sampling, the swab was returned to its original, prelabeled sampling tube for submission to the laboratory. At every selected location, premoistened swabs were collected. Approximately half the sites were also sampled again with unmoistened dry swabs to compare the sampling efficiency of dry swabs to wet swabs and other techniques.

Wipe samples were collected on selected surfaces with a 7.62 x 7.62 cm sterile rayon gauze pad (Dukal Corp., Syosset, NY) premoistened with approximately 5 mL sterile water (Baxter Healthcare Corp., Deerfield, IL). The surface was thoroughly wiped back and forth by using several vertical strokes, folding the exposed side of the pad, and making several horizontal strokes over the same area with the other side of the wipe. The pad was then placed in a prelabeled, 50-mL sterile conical tube and sealed with a cap.

**Figure 2:**
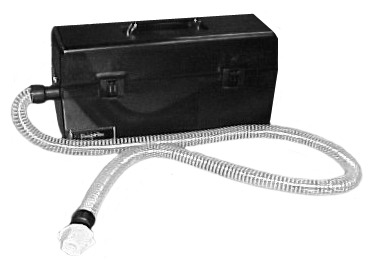
Photograph of HEPA vacuum cleaner and sock sample.

HEPA vacuum sock samples were collected by inserting a cone-shaped filtering trap (dust collection filter sock; Midwest Filtration Co., Fairfield, OH) into the nozzle of a HEPA vacuum cleaner (Atrix International Inc., Burnsville, MN). The vacuum had an electric motor (120 V, 6.6 A, 1 hp) to provide suction of 28 cubic feet (792.4 L) per min through the vacuum nozzle (Figure 2). The plastic sleeve of the dust collection trap was folded over the outside of the nozzle and held in place by hand while the vacuum nozzle was moved slowly back and forth across the sampled surface. The dust collection trap was removed from the vacuum nozzle, placed in a prelabeled, 50-mL sterile conical tube, and sealed with a cap. Before inserting a clean sock into the vacuum nozzle and collecting a subsequent sample, the investigator put on a new pair of gloves and wiped the inside of the vacuum nozzle thoroughly with an alcohol wipe, to physically remove contamination from the nozzle surface (not to sterilize the surface because alcohol does not effectively kill *B. anthracis* spores [10]). To determine whether cross-contamination of subsequent vacuum samples might occur through contamination of the vacuum nozzle during sampling, occasionally a filter sock was inserted into the vacuum nozzle after a sample had been collected and the nozzle cleaned, but the sock was then simply withdrawn and placed in a sterile conical tube for laboratory analysis.

Swab and wipe samples were extracted in a laboratory operated by the USPS contractor at the Brentwood facility. The samples were extracted by adding 20–30 mL 0.3% Tween 20 in PBS to a 50-mL Blue Falcon screw-top tube (Becton Dickinson Labware, Franklin Lakes, NJ) and vortexing the tube for 3 min. The contents of the tube were allowed to settle for 5 min, and swabs and wipes were removed. The tube was centrifuged at 3,000–4,500 rpm, 15–30 min at 10°C, the supernatant removed by decanting, and the pellet was resuspended in 2 mL 0.3% Tween 20 in PBS solution. Approximately half the resuspended extract was shipped to CDC Bioterrorism Surge Capacity and Anthrax Laboratories for culture and confirmatory analysis. The remaining half of the resuspended extract was retained at the laboratory at the Brentwood facility for polymerase chain reaction (PCR) analysis (unpub. data).

At CDC, 0.1 mL of the suspension (approximately 10% of the extract solution) was plated to trypticase soy agar with 5% sheep blood and streaked for quantification. The plates were incubated at 35°C–37°C in ambient air and examined after 24 h and 48 h. Suspect colonies were screened by Level A procedures for identification of *B. anthracis* (11). Identification of all strains was confirmed by standard microbiologic procedures and the Laboratory Response Network (LRN) testing algorithm (12,13). Results of these samples were reported as number of CFUs per plate. To estimate CFUs per sampled surface area, the number of CFUs per plate was multiplied by 20 (2 mL extract solution divided by 0.1 mL plated solution) and divided by the recorded surface area in square centimeters. When the number of colonies on the culture plates exceeded approximately 300, they were reported as too numerous to count.

The HEPA vacuum sock samples were analyzed by an LRN contract laboratory. The HEPA vacuum socks and their contents were weighed on a precision balance. We used the average weight of five unused sock samples to estimate the presampling weight of the vacuum socks; the average weight of the unused socks was 0.70 g (standard deviation 0.02 g). The average weight was subtracted from the postsampling weight of each sock sample to determine the weight of its contents. Approximately 20–30 mL 0.3% Tween 20 in PBS was added to a 50-mL cup containing the sock and its contents and placed on a shaker for 30 min. The contents of the cup were allowed to settle for 5 min; the supernatant then was poured into a 50-mL Blue Falcon screw-top tube (Becton Dickinson Labware). The tube was centrifuged at 3,000–4,500 rpm, for 15–30 min at 10°C; approximately 90% of the starting volume was then removed. The pellet in the bottom of the tube was resuspended in approximately 2 mL 0.3% Tween 20 in PBS, and 0.1 mL (two drops from Pasteur pipette) and 0.01 mL (added by using a calibrated loop) of the suspension were plated to two trypticase soy agar plates with 5% sheep blood and streaked for quantification. The plates were incubated and screened, and suspect colonies were identified by using the same laboratory methods used for the swab and wipe samples. Results of these samples were reported as CFU/g of material collected; the estimated weights of the sock contents were also reported. To estimate CFU per sampled surface area, the reported CFU/g were multiplied by the reported weight of the sock contents and divided by the recorded surface area in square centimeters.

To evaluate the effectiveness of the wipe and HEPA vacuum samples for removing spores from surfaces, at some locations we collected wipe samples over the same surface area previously vacuumed, as well as HEPA vacuum samples over the same surface area previously wiped. We compared the relative difference in CFU/cm^2^ reported for the two methods to evaluate the removal efficiency of the wipe and HEPA vacuum sock samples.

Operations to decontaminate the Brentwood facility had been done since late October 2001 by using HEPA vacuums and sodium hypochlorite solutions. These clean-up operations focused on the DBCS machines. Swab, wipe, and HEPA vacuum sock samples of DBCS machine surfaces that had been cleaned were collected to evaluate the effectiveness of clean-up operations.

PC-SAS computer software was used for all statistical analyses (14). Sample results (positive vs. negative) were analyzed by using simple descriptive statistics, including counts and percents. Agreement between paired sampling methods was assessed by using Cohen’s Kappa, a statistical method that measures agreement beyond what would be expected based on chance alone (15). Kappa scores <0.4 were considered poor agreement, while scores >0.75 indicated excellent agreement; Kappa scores between these values indicated fair to good agreement. Sample levels (CFU/cm^2^) were analyzed by simple descriptive statistics, including sample median and range. Spearman’s rank correlation coefficient significance tests based on ranks that do not assume normality were used as a measure of the association between two paired sampling methods (16). Agreement between paired sampling methods with respect to ordered categories (0, 0.1–1.6, 1.7–15.5, and >15.5 CFU/cm^2^) was assessed by using Kendall’s tau-b statistic, which measures ordinal association (17).

## Results

Descriptive statistics for the culture analysis of the dry and wet swab, wipe, and HEPA vacuum sock samples are shown in Table 1. *B. anthracis* was cultured from 4 (14%) of 28 dry swab samples, while 36 (54%) of 67 wet swabs were culture positive. Fifty-eight (87%) of 67 of the wipe samples and 51 (80%) of 64 of HEPA vacuum sock samples were culture positive. Although CFUs/cm^2^ were reported for each positive sample, these results should only be considered semiquantitative; absolute concentrations cannot be directly compared across the sampling methods. However, the calculated concentrations of *B. anthracis* spores in the culture-positive HEPA vacuum sock samples tended to be greater than in the other types of samples.

**Table 1:** Sample summary statistics for *Bacillus anthracis* culture analysis, Brentwood Mail Processing and Distribution Center, December 17–19, 2001

Method	No. samples tested	*B. anthracis* detected (%)	Range^b^ (CFU/cm^2^)	Median^b^ (CFU/cm^2^)	Level^a^			
					Negative	Low	Medium	High
Dry swab	28	4 (14)	0.45–232.5	60.9	24	1	1	2
Wet swab	67	36 (54)	0.78–232.5	15.5	31	4	14	18
HEPAvacuum	64	51 (80)	0.3–81,000	23.1	13	9	14	28
Wipe	67	58 (87)	0.02–232.5^c^	5.4	9	9	36	13

^a^Level of *B. anthracis* (CFU/cm^2^): negative = 0, low = 0.1–1.6, medium=1.7–15.5, and high=>15.5. ^b^Positive samples only. ^c^232.5 CFU/cm^2^ is the maximum value considered too numerous to count for a concentration; 300 CFU is the maximum value considered too numerous to count for a culture.

None of the blank control samples was positive for *B. anthracis* spores. Of the nine blank HEPA vacuum samples collected from the vacuum nozzle to estimate cross-contamination, eight were culture negative; one *B. anthracis* CFU was detected in one sock.

The results of the dry swab samples are compared with results obtained by using the other types of samples (Table 2). Dry swab samples were collected at 28 locations. These results indicate that when corresponding wipe and HEPA vacuum sock samples were culture positive for *B. anthracis* spores, the dry swab samples detected *B. anthracis* 4 of 23 times. When the corresponding wet swabs were positive for *B. anthracis* spores, the dry swabs detected *B. anthracis* 4 of 13 times. At no time were the dry swabs positive while the other types of corresponding samples did not detect spores. Results of the dry swabs were not included in further comparisons.

**Table 2:** Dry swab versus other sampling methods for 28 locations, Brentwood postal facility, December 17–19, 2001

Method	Dry swab					
	No. concordant samples^a^		No. discordant samples^b^		Correlation	
	Positive (%)	Negative (%)	Dry positive	Dry negative	r_s_^c^	p value^d^
Wet swab	4 (14)	15 (54)	0	9	0.43	0.024
HEPA vacuum	4 (14)	5 (18)	0	19	0.21	0.282
Wipe	4 (14)	5 (18)	0	19	0.07	0.719

^a^Two samples from the same location are concordant if both positive or both negative for *Bacillus anthracis* spores. ^b^Two samples from the same location are discordant if one is positive and the other negative for *B. anthracis* spores. ^c^r_s_ denotes Spearman’s correlation coefficient between level of *B. anthracis* (CFU/cm^2^) obtained by using the dry swab method and the level of *B. anthracis* obtained by using the comparison sampling method. ^d^p value for null hypothesis of zero correlation.

A total of 58 sets of wet swab, wipe, and HEPA vacuum sock samples collected side-by-side were available for comparison, and 67 sets of wet swab and wipe samples collected side-by-side were also available for comparison (Table 3). Results of wet swab and wipe sample analysis were concordant in 64% of the sample comparisons; 23 wipe samples were reported as culture positive when the wet swab samples failed to detect spores, and 1 culture-positive wet swab sample was reported when the corresponding wipe sample was culture negative. Results of the wet swab and HEPA vacuum sock samples were also concordant on 64% of the sample comparisons with similar results as the wet swab and wipe comparison. Twenty-one (36%) HEPA vacuum samples were reported as culture positive when the wet swab samples were negative, but no culture-positive wet swab samples were ever reported when the corresponding HEPA vacuum sock samples were negative. Results of HEPA vacuum sock and wipe samples were concordant 84% of the time; when they were discordant, the corresponding HEPA vacuum sock and wipe samples did not detect *B. anthracis* spores about the same number of times (five negative for HEPA vacuum sock and four negative wipe samples). Only the comparison of HEPA vacuum sock versus wipe sample had a Cohen’s Kappa score >0.4, indicating fair to good agreement (Table 3).

**Table 3:** Comparison of wet swab, wipe, and HEPA vacuum sock sampling methods, Brentwood postal facility, December 17–19, 2001

Methods compared	No. samples	No. concordant samples^a^		No. discordant samples^b^		Cohen’s Kappa
		Positive (%)	Negative (%)	Positive method	Positive method	
Wet swab vs. wipe	67	35 (52)	8 (12)	Wet swab = 1	Wipe = 23	0.24
Wet swab vs. HEPA vacuum	58	27 (47)	10 (17)	Wet swab = 0	HEPA vacuum = 21	0.31
Wipe vs. HEPA vacuum	58	44 (76)	5 (9)	Wipe = 5	HEPA vacuum = 4	0.43

^a^Two samples from the same location are concordant if both positive or both negative for presence of *Bacillus anthracis* spores. ^b^Two samples from the same location are discordant if one is positive and the other negative for presence of *B. anthracis* spores.

The HEPA vacuum sock samples typically collected higher concentrations of *B. anthracis* spores than both the wet swab and wipe samples, and the wipe samples collected higher concentrations of spores than the wet swab samples (Table 4). These comparisons indicate good agreement between the HEPA vacuum sock samples and the wipe samples (Kendall’s tau-b 0.66; Spearman’s rank correlation coefficient 0.81). Although wet swabs were correlated with both the HEPA vacuum samples and the wipe samples, the agreement was not as strong.

**Table 4:** Comparison of *Bacillus anthracis* spore concentration levels in wet swab, wipe, and HEPA vacuum sock samples, Brentwood Mail Processing and Distribution Center, December 17–19, 2001

	HEPA vacuum vs. wet swab		HEPA vacuum vs. wipe		Wet swab vs. wipe	
No. samples	58		58		67	
Levels^a^ agree^b^	22	38%	26	45%	24	36%
Negative	10		5		8	
Low	1		2		0	
Medium	0		8		10	
High	11		11		6	
Levels disagree	36	62%	32	55%	43	64%
Higher levels	34	HEPA vacuum	23	HEPA vacuum	13	Wet swab
Higher levels	2	Wet swab	9	Wipe	30	Wipe
Kendall’s tau-b	0.58		0.66		0.47	
Spearman’s correlation r_s_ (p value)^c^	0.73 (<0.0001)		0.81 (<0.0001)		0.52 (<0.0001)	

^a^Level of *B. anthracis* (CFU/cm^2^): negative = 0, low = 01–1.6, medium=1.7–15.5, and high=>15.5. ^b^Two samples from the same location agree if they are concordant and are both in the same grouping. ^c^p value for null hypothesis of zero correlation.

The randomly selected surface areas where 13 HEPA vacuum sock samples had been collected were immediately sampled again with wipe samples. All the HEPA samples were positive for *B. anthracis* spores ranging in concentrations from 0.5 to 310 CFU/cm^2^. The spore concentrations collected by the subsequent wipe samples (0 to 16 CFU/cm^2^) were usually lower than the original vacuum samples; only two of the subsequent wipe samples were negative for *B. anthracis* spores.

The surface areas where 12 wipe samples were collected, corresponding to 12 of 13 HEPA vacuum sock samples, were immediately sampled again with HEPA vacuum sock samples. All the wipe samples were positive for *B. anthracis* spores, ranging in concentrations from 1.4 to 233 CFU/cm^2^. Only one of the subsequent HEPA vacuum samples was negative for spores and the concentrations in nine of the HEPA vacuum sock samples were virtually the same as on the original wipe samples.

## Discussion

The results of the side-by-side comparison of swab, wipe, and HEPA vacuum sock samples on nonporous surfaces indicated good agreement between the HEPA vacuum sock and wipe samples. However, the HEPA vacuum sock and wipe samples agreed poorly with the swab samples. The wet swabs did not detect spores >33% of the time when spores were detected by the wipe and vacuum sock samples. The dry swabs performed especially poorly, failing to detect spores >66% of the time when spores were detected by wipe and vacuum sock samples. Based on these results, dry swabs should not be used to sample for *B. anthracis* environmental contamination. Applying wet swabs in certain circumstances may be useful, for example, to sample crevices, inside machinery, and places difficult to reach by wipe and HEPA vacuum samples; however, dry swabs should not be used to sample surfaces where wipe and HEPA vacuum samples are likely to yield superior results. Sampling with wipes and HEPA vacuum socks is likely to yield very similar results on nonporous surfaces; wipes are preferable for sampling surfaces with relatively light dust, while HEPA vacuum socks should be selected to sample surfaces with heavy dust. Wipes may become quickly overloaded on dusty surfaces and thus unable to cover a large surface area. The sampling sensitivity of HEPA vacuum socks may be greater because they can collect large dust loads over much larger surface areas than wipes.

The relative difference between the wipe samples and the subsequent HEPA vacuum sock samples was not influenced by the initial concentration of spores collected by the wipe samples. After especially dirty areas were sampled with both wipes and HEPA vacuum sock samples, residual dirt was often still visible.

The samples were collected side by side so that the exact same surface area was not sampled by all methods. Because of nonuniform distribution, spore concentrations may have varied across the surfaces sampled by each method. However, we also set the order of sampling as random, making it unlikely that any particular method consistently encountered fewer spores than the other methods. Strong differences in these particular results more likely resulted from the sampling technique and not to nonuniform distribution of spores on these highly contaminated surfaces, where the different types of samples were collected very close to each other.

In areas likely to have been contaminated over a broad surface at high concentrations (such as DBCS machine no. 17), an adequate number of spores for detection was likely available for all three sampling techniques, but in other, less-contaminated areas, fewer spores were available for detection. Surface sampling clearly has inherent limitations. If investigators are careful to avoid contamination of the samples, the number of false-positive samples is reduced. However, sampling all surfaces within a building is not practical, and some surfaces containing *B. anthracis* spores might be missed.

The measurements collected in this study were not adequate to evaluate the sampling efficiencies of wipe and HEPA vacuum sock samples, particularly since the initial concentrations of spores on the sampled surfaces were unknown. However, sequential HEPA vacuum sock samples indicated better collection efficiency on nonporous surfaces than wipe samples. This efficiency is evident because wipe samples collected following vacuum samples were much lower than the initial vacuum samples, while the vacuum samples collected after wipe samples often collected a similar concentration of spores as the initial wipe samples. Care was taken after sampling to stay within the previously sampled area, but spores from outside the previously sampled area may have been inadvertently collected by the HEPA vacuum samples (e.g., spores from surrounding unsampled areas may have been drawn into the HEPA vacuum sock).

To avoid contamination of the vacuum when collecting samples, using disposable inserts may be more appropriate, such as cardboard sleeves, which can be placed inside the vacuum nozzle; the sampling sock can then be inserted into the sleeve and discarded after sampling. These sleeves should be discarded after sampling. Disposable inserts may prevent cross-contamination of the vacuum nozzle or subsequent sock samples. Care must be taken to prevent contamination of the inserts before they are used for sampling. While vacuum nozzles may not always be completely cleaned after sampling, our investigation indicated that cross-contamination could not be the reason for the high concentrations of spores detected on the numerous HEPA vacuum sock samples.

The results of this investigation may be used to guide future sampling efforts and serve as a baseline for follow-up measurements after the building has been cleaned further. The sampling and analytical techniques used in our study may provide useful reference for evaluations of other situations in the future. This study provides additional evidence for the need to quantify sampling efficiency to develop the type of limit-of-detection data normally created for other types of sampling and analytical methods. The collection efficiency (removing spores from the surface) and recovery efficiency (removing spores from the sampling media) need to be further evaluated for these methods. Our study focused on sampling nonporous surfaces; under these circumstances, HEPA vacuum sock samples and wipe samples performed similarly. However, this level of agreement may be difficult to achieve in sampling porous materials such as carpet and furniture, and the collection efficiency of sampling methods on other surfaces needs to be evaluated. Understanding the sampling efficiency of these methods on various types of surfaces is a critical requirement for future efforts to develop numerical criteria for surface contamination and potential exposures to humans. Lack of understanding about the efficiency of various sampling methods limits our ability to determine whether an environment has been adequately cleaned.

## References

[1] Centers for Disease Control and Prevention. Evaluation of *Bacillus anthracis* contamination inside the Brentwood Mail Processing and Distribution Center B District of Columbia, October 2001. MMWR Morb Mortal Wkly Rep. 2001;50:1129–33. 11824387

[2] Angelotti R, Foter M, Busch K, Lewis K. A comparative evaluation of methods for determining the bacterial contamination of surfaces. Food Res. 1958;23:175–85.

[3] Angelotti R, Wilson J, Litsky W, Walter W. Comparative evaluation of the cotton swab and Rodac methods for the recovery of *Bacillus subtilis* spore contamination from stainless steel surfaces. Health Lab Sci. 1964;1:289–96. 4964105

[4] Favero M, McDade J, Robertson J, Hoffman R, Edwards R. Microbiological sampling of surfaces. J Appl Bacteriol. 1968;31:336–43. 4880687 10.1111/j.1365-2672.1968.tb00375.x

[5] Whitfield W, Beakley J, Dugan V, Hughes L, Morris M, McDade J. Vacuum probe: new approach to the microbial sampling of surfaces. Appl Microbiol. 1969;17:164–8. 4975452 10.1128/am.17.1.164-168.1969 PMC377631

[6] Kirschner L, Puleo J. Wipe-rinse technique for quantitating microbial contamination on large surfaces. Appl Environ Microbiol. 1979;38:466–70. 394682 10.1128/aem.38.3.466-470.1979 PMC243518

[7] Buttner M, Cruz-Perez P, Stetzenbach L. Enhanced detection of surface-associated bacteria in indoor environments by quantitative PCR. Appl Environ Microbiol. 2001;67:2564–70. 10.1128/AEM.67.6.2564-2570.2001 11375164 PMC92908

[8] Dull P, Wilson K, Kournikakis B, Boulet C, Ho J, Ogston J, Risk of *Bacillus anthracis* aerosolization associated with a contaminated mail sorting machine. Emerg Infect Dis. 2002;8:1044–7. 12396913 10.3201/eid0810.020356 PMC2730297

[9] Centers for Disease Control and Prevention. Comprehensive procedures for collecting environmental samples for culturing *Bacillus anthracis*. Available from: URL: http://www.bt.cdc.gov/Agent/environmental-sampling-apr2002.asp. Accessed May 10, 2002.

[10] Alcamo I. Fundamentals of microbiology. Sudbury, Massachusetts: Jones and Bartlett Publishers; 2000. p. 710–1.

[11] Centers for Disease Control and Prevention. American Society for Microbiology, Association of Public Health Laboratories. Basic diagnostic testing protocols for Level A laboratories for the presumptive identification of *Bacillus anthracis.* Available from: URL: http://www.asmusa.org/pcsrc/ban.asm.la.cp.102401f.pdf . Accessed October 2001.

[12] Logan N, Turnbull P. Bacillus and recently derived genera. In: Murray P, Baron E, Pfaller M, Tenover F, Yolken R, editors. Manual of clinical microbiology. 7th edition. Washington, D.C.: American Society for Microbiology; 1999. p. 357–69.

[13] Khan A, Morse S, Lillibridge S. Public health preparedness for biological terrorism in the USA. Lancet. 2000;356:1179–82. 10.1016/S0140-6736(00)02769-0 11030310

[14] SAS Institute, Inc. SAS/STAT user’s guide, version 8. Cary, NC: SAS Institute, Inc.; 1999. p. 3884.

[15] Fleiss JL. Statistical methods for rates and proportions. 2nd edition, New York: John Wiley & Sons, Inc.; 1981. p. 218.

[16] Hollander M, Wolfe DA. Nonparametric statistical methods. 2nd edition. New York: John Wiley & Sons, Inc.; 1999. p. 394–408.

[17] Blalock H. Social statistics. New York: McGraw-Hill Co.; 1979. p. 436–9.

